# Reconstitution of Mitochondria Derived Vesicle Formation Demonstrates Selective Enrichment of Oxidized Cargo

**DOI:** 10.1371/journal.pone.0052830

**Published:** 2012-12-26

**Authors:** Vincent Soubannier, Peter Rippstein, Brett A. Kaufman, Eric A. Shoubridge, Heidi M. McBride

**Affiliations:** 1 Montreal Neurological Institute, McGill University, Montréal, Québec, Canada; 2 Lipoproteins and Atherosclerosis Group, University of Ottawa Heart Institute, Ottawa, Ontario, Canada; 3 Department of Animal Biology, School of Veterinary Medicine, University of Pennsylvania, Philadelphia, Pennsylvania, United States of America; Boston University, United States of America

## Abstract

The mechanisms that ensure the removal of damaged mitochondrial proteins and lipids are critical for the health of the cell, and errors in these pathways are implicated in numerous degenerative diseases. We recently uncovered a new pathway for the selective removal of proteins mediated by mitochondrial derived vesicular carriers (MDVs) that transit to the lysosome. However, it was not determined whether these vesicles were selectively enriched for oxidized, or damaged proteins, and the extent to which the complexes of the electron transport chain and the mtDNA-containing nucloids may have been incorporated. In this study, we have developed a cell-free mitochondrial budding reaction *in vitro* in order to better dissect the pathway. Our data confirm that MDVs are stimulated upon various forms of mitochondrial stress, and the vesicles incorporated quantitative amounts of cargo, whose identity depended upon the nature of the stress. Under the conditions examined, MDVs did not incorporate complexes I and V, nor were any nucleoids present, demonstrating the specificity of cargo incorporation. Stress-induced MDVs are selectively enriched for oxidized proteins, suggesting that conformational changes induced by oxidation may initiate their incorporation into the vesicles. Ultrastructural analyses of MDVs isolated on sucrose flotation gradients revealed the formation of both single and double membranes vesicles of unique densities and uniform diameter. This work provides a framework for a reductionist approach towards a detailed examination of the mechanisms of MDV formation and cargo incorporation, and supports the emerging concept that MDVs are critical contributors to mitochondrial quality control.

## Introduction

Mitochondrial quality control is an essential process required to clear the accumulation of unfolded, oxidized, or otherwise damaged proteins and lipids from the organelle. As the “energy powerhouse”, this organelle, perhaps more than any other, depends upon a series of pathways that continually survey for damage. Emerging evidence highlights the redundancies intrinsic to mitochondrial quality control, with at least four distinct mechanisms of turnover. The first characterized pathway is the constitutive proteolysis of unfolded and oxidized proteins within the matrix and intermembrane space. Proteases of the AAA ATPase family are localized on both sides of the inner membrane and play essential roles in the degradation of unfolded or unassembled proteins, which can be triggered by protein oxidation [1]. In the yeast *Saccharomyces cerevisiae* it has been shown that protease dependent turnover of misfolded and oxidized proteins reaches 6–12% of the total mitochondrial protein per hour [2], [3]. This is the only pathway whose contribution to mitochondrial turnover has been quantified, and is generally considered to be the most robust of all the pathways. On the other hand, following fragmentation, some of these small mitochondria may by functionally compromised, which is demonstrated by an inability to restore or maintain an electrochemical gradient [4]. Loss of a proton electrochemical gradient leads to the activation of an inner membrane protease Oma1, which cleaves the inner membrane fusion GTPase Opa1, further isolating the organelle from the mitochondrial reticulum [5], [6]. The diminuation of electrochemical potential will eventually lead to the recruitment of the ubiquitin E3 ligase Parkin to the surface, which finally delivers the failed organelle to the autophagosome [7]. A great deal of new work has been done in the past few years focusing on the molecular mechanisms that govern selective mitophagy, and the implications of these pathways in neurodegenerative disease. These studies have highlighted the importance of mitochondrial quality control in cell survival. Importantly, the contribution of mitophagy in steady state mitochondrial protein turnover has been difficult to quantify [8], which has led to some confusion concerning the role of mitophagy in tissues and primary neuronal cultures [9], [10], [11], [12], [13]. The emerging evidence supports a role for Parkin-mediated mitophagy in primary neurons, however the kinetics of clearance can be significantly longer and more complex in primary neuronal cultures, for example, following 24 hours of treatment with high doses (10 µM) of a protonophore CCCP.

In addition to mitochondrial proteolysis and mitophagy, mitochondrial membrane proteins can be ubiquitinated and targeted to the proteasome. This process has been shown to mainly function in the regulated turnover of individual proteins like MULE [14], [15], the yeast Fzo1 [16] and the inner membrane uncoupling protein, UCP2 [17]. This may not require specific damage, rather cellular cues like mitosis or cell death can initiate selective protein removal from the organelle. However, upon global mitochondrial uncoupling with protonophores, the cytosolic ubiquitin E3 ligase Parkin is recruited to the mitochondria along with p97/VCP, which together ubiquitinate and target outer membrane proteins to the proteasome [18], [19], [20], [21], [22]. The identification of these machineries continues to provide mechanistic insights into mitochondrial membrane associated degradation (OMMAD) [23].

We have recently documented the ability of mitochondria to generate mitochondrial derived vesicles (MDVs) that selectively transport mitochondrial proteins to either the peroxisomes or the lysosomes [24], [25], [26], [27]. MDVs are formed from functionally intact, respiring mitochondria in a manner independent from the fission GTPase DRP1. Ultrastructural analysis of cells has shown budding profiles on the mitochondrial surface consistent with vesicle formation [25], [26], [27]. Most critically, MDVs show evidence of cargo selectivity, where, for example, some vesicles may carry an outer membrane protein MAPL, but lack a second outer mitochondrial membrane protein Tom20. Initially we characterized a number of distinct MDV pools in the cell and we determined that one population of MDVs target the peroxisomes [25]. The functional importance of protein delivery to peroxisomes through this pathway has not yet been determined. More recently we reported that another class of MDVs are generated as an early response to oxidative stress [27]. These MDVs are then addressed to the lysosomes for degradation, a process that does not involve the core autophagy machinery. Importantly, even in the absence of any stress trigger, addition of lysosome inhibitors led to a 5–10 fold accumulation of MDVs within the cell [27]. This highlighted the fact that MDV transport to the late endosome is constitutively active, and has the capacity to selectively remove proteins, complexes and lipids from actively respiring mitochondria.

Here we extend our previous work and describe a cell-free assay that reconstitutes MDV formation. Using this tool, we demonstrate that the cargo incorporated into MDVs are selectively enriched for oxidized proteins. We also perform an analysis of the cargo that is incorporated into MDVs, revealing a tight correlation between the nature of the damage trigger and the identity of the cargo. The specificity was further demonstrated by the exclusion of several OXPHOS complexes as well as mtDNA from the MDVs generated *in vitro*. Electron micrographs of MDVs separated by sucrose density centrifugation revealed the formation of both single and double membrane structures, with a uniform diameter and similar to those observed in cells or *in vivo*. With this, we demonstrate that MDVs comprise a quantitative and highly selective pathway utilized for mitochondrial quality control, providing a platform for future research.

## Materials and Methods

### Ethics Statement

Fresh bovine heart was obtained with permission from a government-licensed abattoir in St. Albert, Ontario (Joe Savage Abattoir at 113 Principale, St.Albert, Ontario K0A 1M0).

### Antibodies and Reagents

Antibodies were purchased from the following providers: anti-Core2, anti-VDAC, anti-Cox1, anti-PDH, anti-NDUFA6, anti-NDUFS3, anti-su30kD, and anti-F1 β were all obtained from Mitosciences (Eugene, Oregon, USA); anti-aconitase was purchased from Santa Cruz Biotechnology and anti-TFAM has been previously described [28].

### Purification of Mitochondria and cytosol from Bovine Heart

Fresh cow heart was cut into small pieces and washed a number of times in ice cold PBS. The volume equivalent of heart tissue was measured and then homogenized with 2 times the tissue volume of Mitochondrial Isolation Buffer (MIB; 220 mM mannitol, 68 mM sucrose, 80 mM KCl, 0.5 mM EGTA, 2 mM magnesium acetate, 20 mM Hepes pH 7.4) and in presence of protease inhibitor cocktail (Roche Diagnostics), using quick pulses in a Waring blender. The lysate was centrifuged twice at 3,000×g at 4°C for 20 minutes to remove nuclei and unbroken cells. The supernatant was then centrifuged at 10,000×g for 20 minutes at 4°C. The supernatant was kept for cytosol preparation while the pellet was washed with 500 mL of MIB buffer and centrifuged again at 10,000×g for 20 minutes at 4°C. The mitochondrial pellet was finally re-suspended in a minimum volume of MIB buffer and the protein concentration was determined by Bradford assay (Biorad).

For the preparation of cytosol, the first mitochondrial supernatant fraction (from above) was subjected to further centrifugation steps at 4 degrees Celsius in order to remove remaining intracellular organelles. The supernatants containing cytosolic proteins were snap frozen in liquid nitrogen. Prior to use in the assay, cytosols were thawed and subjected to centrifugation at 180,000×g and the protein concentrations were determined (generally ∼ 20 mg/ml).

### Reconstitution of MDV formation in vitro

Five milligrams of purified cow heart mitochondria were washed 3 times in 1 mL of MIB (220 mM mannitol, 68 mM sucrose, 80 mM KCl, 0.5 mM EGTA, 2 mM magnesium acetate, 20 mM Hepes pH 7.4) by centrifugation at 10,000×g at 4°C. The first wash was performed in presence of protease inhibitor cocktail (Roche Diagnostics). The washed mitochondria were then incubated in the budding reaction in 500 μl of an osmotically controlled, buffered environment including an energy regenerating system, where the final concentrations of reagents were: 220 mM mannitol, 68 mM sucrose, 80 mM KCl, 0.5 mM EGTA, 2 mM magnesium acetate, 20 mM Hepes pH 7.4, 1 mM ATP, 5 mM Succinate, 80 μM ADP, 2 mM K2HPO4, pH 7.4. Volumes were controlled using concentrated stock solutions to ensure a controlled osmolarity in each reaction. Following the reaction, the intact mitochondria were removed from the mixture by two sequential centrifugations at 10,000×g at 4C. The supernatants containing the MDV fraction were treated with 0.5 mg/ml trypsin for 10 minutes at 4C, unless prepared for the sucrose density centrifugation. Following trypsin treatment, loading buffer was added and the samples were separated by SDS-PAGE, transferred to nitrocellulose membranes and blotted.

We tested the effect of a panel of damage by adding to the assay either: 50 μM Antimycin A, 50 μg/ml chloramphenicol, 100 μM GTPγS, 125 μM oligomycin, 200 µM xanthine and 0.4U xanthine oxidase, 20 mM NEM, all from Sigma.

### Sucrose gradient fractionation

All steps were carried out at 4°C. The supernatants obtained from budding reactions were adjusted to 40% in mitochondrial isolation buffer (220 mM mannitol, 68 mM sucrose, 80 mM KCl, 0.5 mM EGTA, 2 mM magnesium acetate, 20 mM Hepes pH 7.4) and loaded on the bottom of a discontinuous sucrose gradient with steps at 40%, 30%, 20%, and buffer. Samples were subjected to centrifugation at 150,000×g for 6 hours and fractions were collected for analysis as indicated.

### Oxyblot

Protein oxidation levels were monitored using the oxyblot™ protein oxidation detection kit by chemicon International, following manufacturers instructions. Protein concentration of supernatants containing the MDV fraction was determined by Bradford assay. 20 μg of supernatant were solubilized in 6% SDS with 50 mM DTT in a final volume of 100 μl. Samples were then incubated with 10 μl of 1X DNPH solution for 15 minutes at room temperature (or with 10 μl of 1X derivatization-control solution for the negative controls). The reaction was quenched with 7.5 μl of neutralization solution in all tubes. Following neutralization treatment, loading buffer containing 2-mercaptoethanol was added and the samples were separated by SDS-PAGE, transferred to nitrocellulose membranes and blotted. Membranes were blocked for 1 h in the blocking/dilution buffer (1% BSA in PBS at pH 7.4 with 0.05% Tween-20). Membranes were then incubated for 1 h at room temperature with the anti-DNP primary antibody diluted at 1∶150 in blocking/Dilution buffer just before use. Membranes were washed with PBS-Tween (15 minutes, then twice for 5 minutes). Membranes were then incubated for 1 h at room temperature with the secondary antibody diluted at 1∶300 in blocking/Dilution buffer just before use. Finally, membranes were washed as above before the incubation with ECL.

### mtDNA extraction

800 μl of vesicles were isolated from the budding assay above and divided in half. The first half was treated by trypsin and analyzed by western blot to estimate protein cargo selection. To estimate the mtDNA content of the second half, calcium concentration was first adjusted to 2 mM. The supernatants were then incubated 20 min at 4°C in presence of 10 U of DNAse I to degrade unprotected mtDNA. The reaction was quenched by using 15% TCA and the precipitate was pelleted by centrifugation at 17,000×g for 5 min. The pellet was re-suspended in 400 μl of 100 mM Tris pH 8.5, 0.2% SDS, 5 mM EDTA, 200 mM NaCl, and incubated overnight with 10 μl of Qiagen proteinase K solution in a 55°C waterbath. Then, 50 μl of 4 M potassium acetate at pH 4.5–5 was added and the solution was incubated for 10 min on ice, followed by centrifugation at 17,000×g for 5 min. Finally, the supernatant were submitted to isopropanol precipitation and the pellet was used as a template for PCR reaction using primer recognizing bovine CoxI sequence (CoxI-FWD 5′-CCAAGATGCAACATCACCAA-3′; CoxIREV 5′-CTGGGATTGCGTCTGTTTTT-3′).

### Electron Microscopy

Isolated mitochondria from bovine heart and the supernatant resulting from the *in vitro* mitochondrial budding reaction were fixed in 1.6% glutaraldehyde in PBS pH 7.4 prior to centrifugation at 10,000×g or 200,000×g, respectively. The fixed samples were resuspended in 0.3% low-melting agarose, recentrifuged and the resulting pellet cut into 1 mm pieces. Samples were then postfixed in 1% osmium tetroxide in 0.1 M Na cacodylate buffer, *en bloc* stained in 3% aqueous uranyl acetate, dehydrated in ascending ethanol and embedding in Spurr epoxy resin and processed as previously described (Neuspiel et al., 2008).

## Results

### Reconstitution of MDV formation in vitro

**Figure 1:**
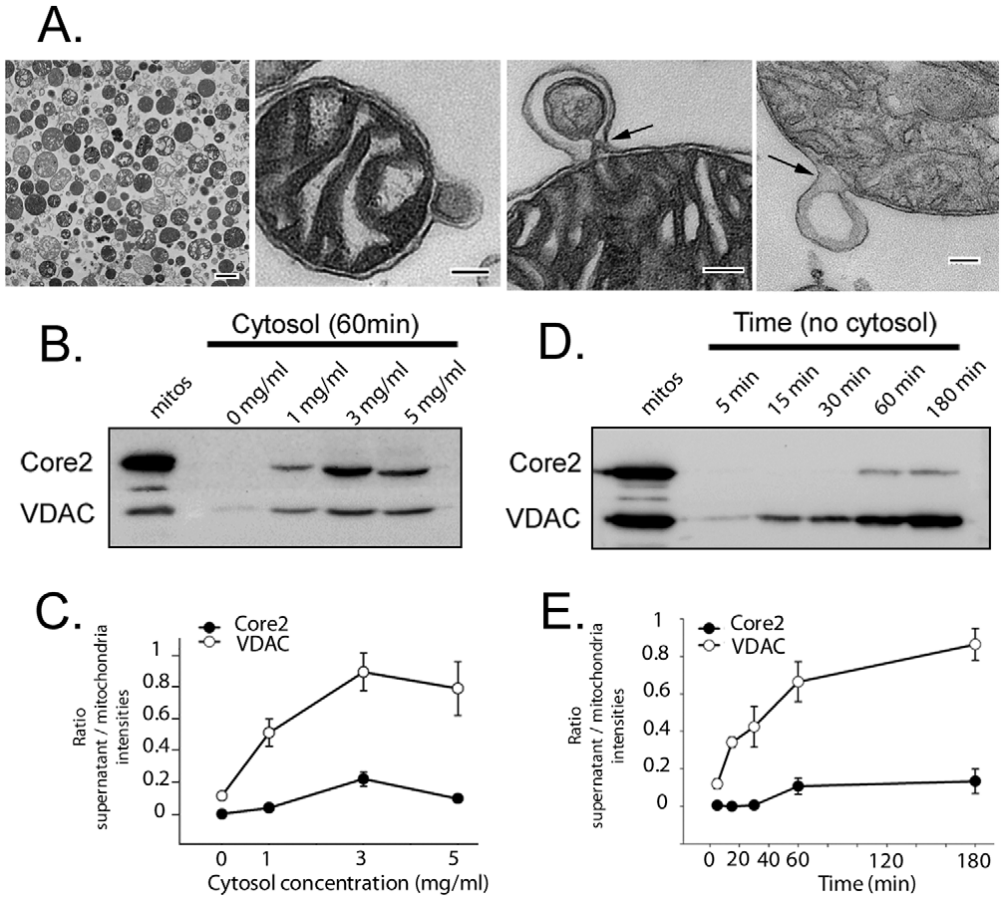
Reconstitution of MDV formation in vitro. A) EM sections of isolated mitochondria from bovine heart showing vesicular profiles. Scale bars are 500 nm in first panel, and 100 nm in 3 panels highlighting potential MDVs. B) Titration of cytosol with 5 mg of isolated mitochondria at 37°C for 60 minutes in the presence of an ATP regenerating system. Mitochondria were removed by centrifugation and 10% of the supernatant loaded onto an SDS-PAGE gel for transfer and western blotting, as indicated. 20 μg of starting mitochondria were loaded as an input control. C) The relative intensities of the cargo within the supernatant were quantified relative to that found within the 20 μg of starting mitochondria. Standard errors were calculated from three different gels loaded with the same reaction. The data is representative of at least 3 independent experiments. D) Time course of MDV formation under minimal conditions in the absence of cytosol. The reaction was incubated at 37°C for the indicated times in the presence of an ATP regenerating system and the supernatants were separated by SDS-PAGE and transferred for western blots as shown. E) Quantification of D), as in C).

To determine the mechanism of MDVs formation and cargo incorporation, we reconstituted the reaction from purified bovine heart mitochondria. It has long been established that mitochondria isolated from heart tissue are highly pure, and are functionally robust within cell-free assays [29], [30]. We first tested whether mitochondria isolated from heart were able to generate MDVs by embedding the glutaraldehyde-fixed mitochondria within low melt agarose prior embedding and sectioning for ultrastructural analysis. Indeed, heart mitochondria revealed the presence of 60–100 nm vesicular structures still attached to a few mitochondria (Figure 1A). In most cases, the presence of both membranes is clearly visible, often with a constriction site evident (Figure 1A, third panel, arrow). We also observed the outer membrane forming protrusions with constrictions at their base, consistent with the formation of single membrane bound vesicles (Figure 1A right panel, arrow). The presence of these structures suggested that bovine heart mitochondria were an appropriate source for the development of an *in vitro* reconstitution assay.

We developed the cell-free budding assay based on established systems used in the earlier ER budding assay systems [31], [32]. Purified mitochondria were incubated for 60 min at 37°C in the presence of cytosol and an energy regenerating mixture. The buffer systems, concentrations of mitochondria used, and time scale of these reactions were established previously in the development of mitochondrial protein import assays [29], [33]. Following the reaction, intact mitochondria were separated by centrifugation at 8,000×g for 20 minutes. The post-mitochondrial supernatant was then treated with trypsin to remove any background signal resulting from broken mitochondria. The trypsin-resistant proteins incorporated within MDVs were examined by Western blot. The assay was stimulated by the presence of cytosol, with peak activity at 3 mg/ml cytosol (Figure 1B, C), and was time-dependent (Figure 1D, E). To quantify the relative enrichment of each mitochondrial protein within the supernatant fractions, we compared the signal intensity relative to that found in the initial isolated mitochondrial pellet. The data reveal that VDAC (ratio of 0.9) is more enriched in vesicles compared to the complex III subunit Core 2 (ratio of 0.2) (Figure 1C). The differential enrichment of cargo found within the supernatant fraction provides evidence for cargo selectivity in this *in vitro* reconstitution assay.

### Mitochondrial stress induces MDV formation in vitro

**Figure 2:**
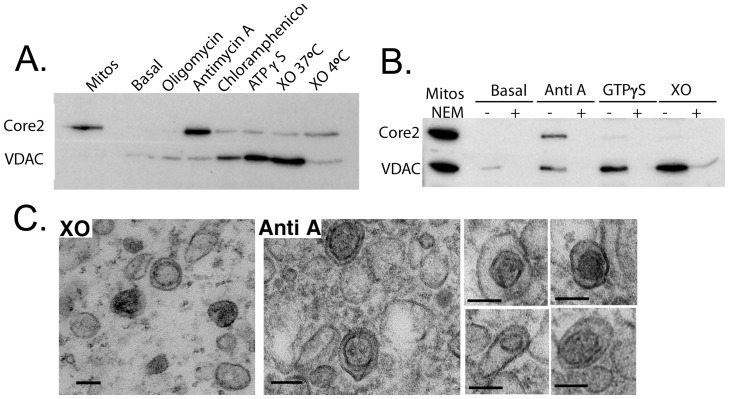
Mitochondrial stress triggers MDVs in vitro. A) Isolated mitochondria were incubated in the absence of cytosol, with an energy regenerating system, for 60 minutes at 37°C (or 4°C) under the indicated conditions: 10 µM oligomycin, 50 µM Antimycin A, 50 µM Chloramphenical, 0.1 mM ATPγS, 200 µM xanthine and 0.4U xanthine oxidase. Along with 2 µg of the starting mitochondria, supernatants of the reactions were separated by SDS-PAGE for analysis by western blot, probing for anti-Core 2, a component of mitochondrial electron transport chain complex III, and the outer membrane channel VDAC. At the exposures shown here, the VDAC western blot does not detect the signal within the starting mitochondria. The data is representative of at least 3 independent experiments. B) As in A) except that 20 mM of NEM was added when indicated, and 0.1 mM GTPγS was used where indicated. C) Supernatants obtained from reactions performed in the presence of either 200 µM xanthine and 0.4U xanthine oxidase or 50 µM Antimycin A were fixed, and isolated in low-melt agarose and prepared for EM analysis. Images show thin sections from the pelleted MDVs, revealing both single and double-membrane bound vesicles. Note the differential electron electron density of material within the inner membrane compared to the space between the two membranes. Scale bars are all 100 nm.

Our previous work has demonstrated that MDVs are stimulated during oxidative stress [27]. To validate our *in vitro* assay, we therefore examined whether vesicle formation can be stimulated *in vitro* upon a panel of various mitochondrial stressors (Figure 2A). Incubation without drug treatment did not result in a significant signal for either inner membrane Core 2 or the outer membrane channel VDAC. Addition of the complex V ATP synthase inhibitor did not increase MDV formation, however addition of the complex III inhibitor Antimycin A led to a significant increase in the appearance of protease-resistant Core 2 protein within the supernatant fraction, without a concomitant increase in VDAC (Figure 2A). Poisoning the mitochondrial ribosomes with chloramphenicol, or addition of ATPγS, both increased the presence of VDAC within the supernatant fraction, yet Core 2 was not enriched. Xanthine oxidase /Xanthine (XO/X) was used as an external ROS generator which led to a temperature-dependent enrichment of VDAC within MDVs lacking Core2. These data are consistent with our previously published evidence demonstrating that oxidative damage triggers the generation of MDVs [27]. However, the data further revealed that the type of oxidative stress determined the identity of the cargo incorporated within the MDVs. Antimycin A inhibits the Core2-containing complex III, suggesting that local ROS generated within the complex may have triggered it's selective incorporation into MDVs. In contrast, general ROS within the reaction, or the presence of ATPγS or chloromphenical led to VDAC-specific inclusion into the supernatant fraction, without the incorporation of Core2. Since mitochondria generate their own ATP within this assay system, we consider that non-hydrolyzable ATP may induce mitochondrial stress rather indirectly.

A hallmark feature of MDV formation is the independence from the mitochondrial fission GTPase Drp1. To test whether MDV formation *in vitro* is independent upon GTP hydrolysis, we incubated the budding reaction in the presence of a non-hydrolyzable analogue of GTP, GTPγS (Figure 2B). In this reaction, we again observe the increase in Core2 incorporation into MDVs in the presence of Antimycin A. Addition of GTPγS led to an increase in the presence of VDAC within the supernatant fractions, comparable to the Xanthine Oxidase/Xanthine stimulation (Figure 2B). This demonstrates that GTP hydrolysis is not essential to constrict or pinch the vesicles from the mitochondria, consistent with the independence from Drp1. In this experiment, we included a non-specific alkylator of free cysteine residues, N-ethylmaleimide. In all conditions, MDV formation was completely abolished in the presence of NEM. NEM sensitivity is a hallmark feature of cell-free membrane transport assays and indicates the requirement for an enzyme containing free thiol groups in the budding process [34]. In addition, high concentrations of NEM have been shown to open the mitochondrial permeability transition pore, which can occur in swelling, or damaged mitochondria [35]. Therefore, the generation of MDVs does not correlate with PTP opening, providing further support for the specificity of the reaction.

We further examined the MDVs within the supernatant of XO/X (Figure 2C left panel) or Antimycin (Figure 2C right panels) treated mitochondria by electron microscopy. In both cases we observe both single and double membrane bound vesicular profiles, between 70–120 nm in diameter. In the case of Antimycin A treatment, many of these structures contained electron dense inner core vesicular structures that were devoid of any cristae or further invaginations (Figure 2C). These images were consistent with what we have previously observed by EM within intact cells [25], [26], [27], and further highlight the purity and intactness of our cell-free reactions.

### Stress-induced MDVs generated are enriched in oxidized cargo

**Figure 3:**
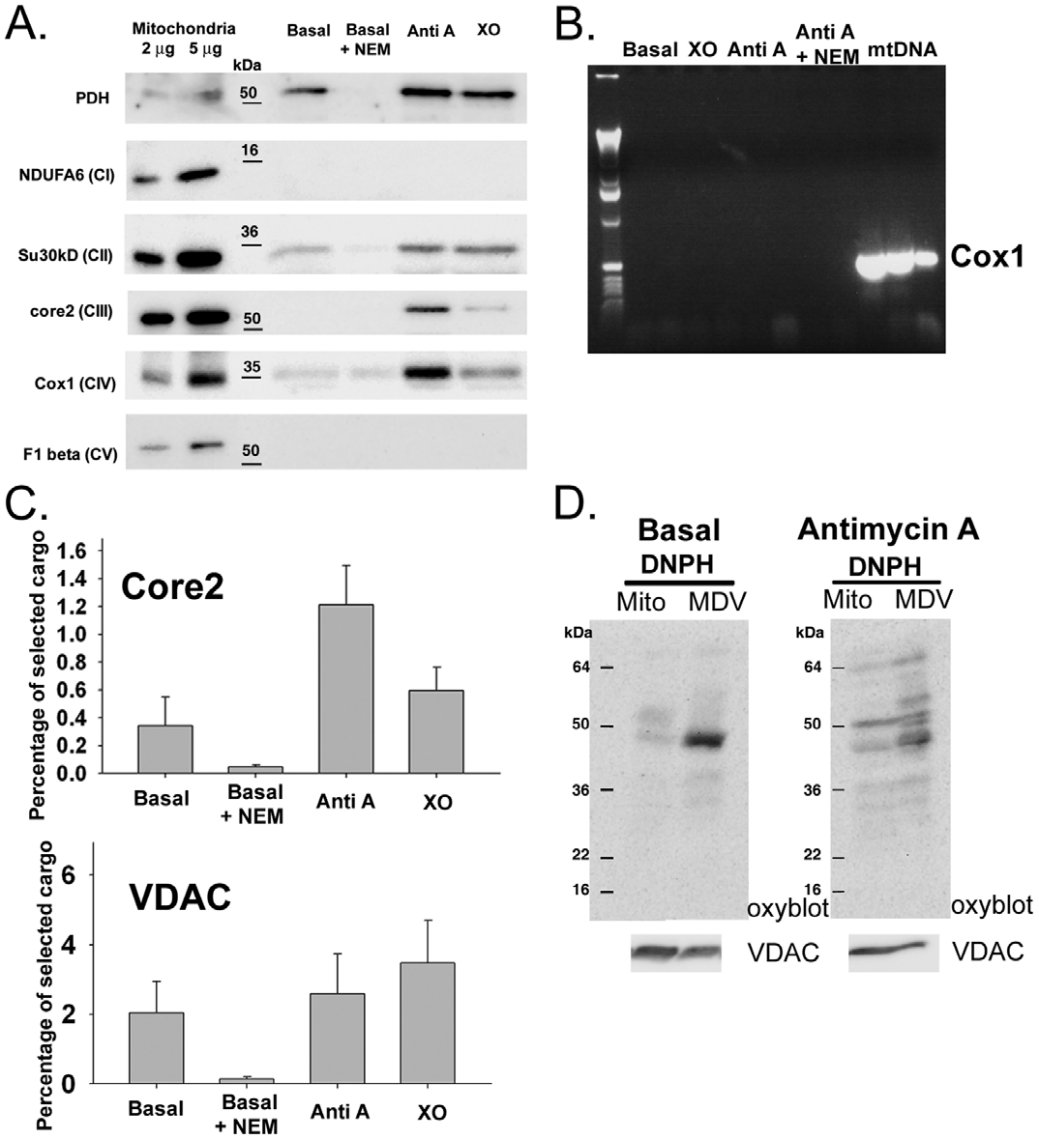
Stress-induced MDVs generated in vitro are enriched in oxidized cargo. A) Reactions were performed as indicated and supernatants were separated by SDS-PAGE and transferred for western blots, including the matrix enzyme pyruvate dehydrogenase (PDH), and the indicated components of the electron transport chain complexes I through V. Note the absence of subunits within complexes I and V. B) Reactions were done as in B) and extraction of mtDNA was performed as described in materials and methods, and used as a template in a PCR reaction amplifying the mitochondrial DNA encoded bovine Cox1 gene. The starting mitochondria were used as a positive control, which efficiently amplified the Cox1 sequence, however there was no incorporation of mtDNA under any of the conditions tested. C) Normalizing the relative immunoreactivity of each cargo within the starting mitochondria (loading 2 and 5 µg), we quantified the amount of protein released into the trypsin-resistant supernatant fractions under the conditions indicated. Quantification of the indicated cargo was done from at least 3 independent experiments (error bars: SE). D) Reactions were performed under basal conditions, or upon treatment with Antimycin A as in A) except that the supernatants were not submitted to trypsin treatment. Free carbonyl groups within the fractions were derivitized with DNPH and quenched prior to separation by SDS-PAGE and blotting with an anti-DNPH Oxyblot antibody to reveal the oxidized proteins. The detection of protein oxidation from 20 µg material is shown, and VDAC was probed as a loading control for mitochondria and MDVs.

To further characterize the cargo incorporated into MDVs under oxidative stress, we tested for the presence of multiple proteins within the trypsinized post-mitochondrial supernatants of various treatments (Figure 3A). Although components of OXPHOS complexes II, III and IV are differentially incorporated within these MDVs under various treatments, proteins of complex I and V were excluded (Figure 3A). We also tested a second component of complex I, NDUFS3, and did not see incorporation within MDVs (Figure S1A). A protein known to be readily oxidized, aconitase, was also absent, consistent with its efficient degradation through the protease system [3] (Figure S1A). As seen previously by confocal imaging [27], the matrix enzyme pyruvate dehydrogenase was also enriched in MDVs upon the induction of damage, indicating that soluble matrix cargo is also incorporated (Figure 3A). We probed the reactions with antibodies against the nucleoid protein TFAM (Figure S1B) [28], and performed PCR reactions to amplify mtDNA within the supernatants. Under the conditions examined, we found no evidence that mtDNA is incorporated within MDVs (Figure 3B).

Given the nature of the cell free assay system where we can quantify the protein within the starting mitochondria, we were able to quantify the relative percent of the proteins incorporated within the vesicles (Figure 3C). Using data from at least 3 independent experiments, we observe ∼1.2% of Core 2 is incorporated within MDVs per hour upon incubation with Antimycin A (Figure 3C, upper panel). Core 2 levels are increased slightly upon treatment with XO, but the enrichment is most evident upon inhibition of complex III with Antimycin A treatment. In contrast, VDAC is seen to incorporate between 2–4% of total protein per hour, with the highest levels seen upon incubation with XO (Figure 3C, lower panel). It is likely that these numbers under-represent the actual amount of protein turned over by MDVs since cell free assays are typically not very efficient. With this in mind, we consider that MDV transport carries a significant amount of mitochondrial protein for turnover. In comparison, mitochondrial proteases are known to degrade between 6–12% of total proteins per hour, which is within the range of MDV incorporation [2]. Moreover, the steady-state turnover of mitochondrial proteins through MDVs is supported by our previous observation that inhibition of lysosomal activity leads to a 5–10 fold accumulation of MDVs within the cytosol, even in absence of acute oxidative stress [27].

Given the stimulation of MDV formation upon oxidative stress, we tested whether there is enrichment for oxidized protein within the cargo. The isolated MDVs were incubated in presence of DNPH to derivatize free carbonyl groups, a signature of oxidation, which were then detected by Western blot analysis using antibodies raised against DNPH (Figure 3D). Loading equivalent protein amounts of mitochondria and vesicle fractions, the data show that the MDVs are selectively enriched in oxidized cargo relative to the starting mitochondria, both in the basal conditions (Figure 3D, left panel), and upon treatment with Antimycin A (Figure 3D, right panel). Taken together, the reconstitution experiments demonstrate that MDVs are stimulated under conditions of stress and are selectively enriched for oxidized cargo.

### Ultrastructural analysis of MDVs generated in vitro

**Figure 4:**
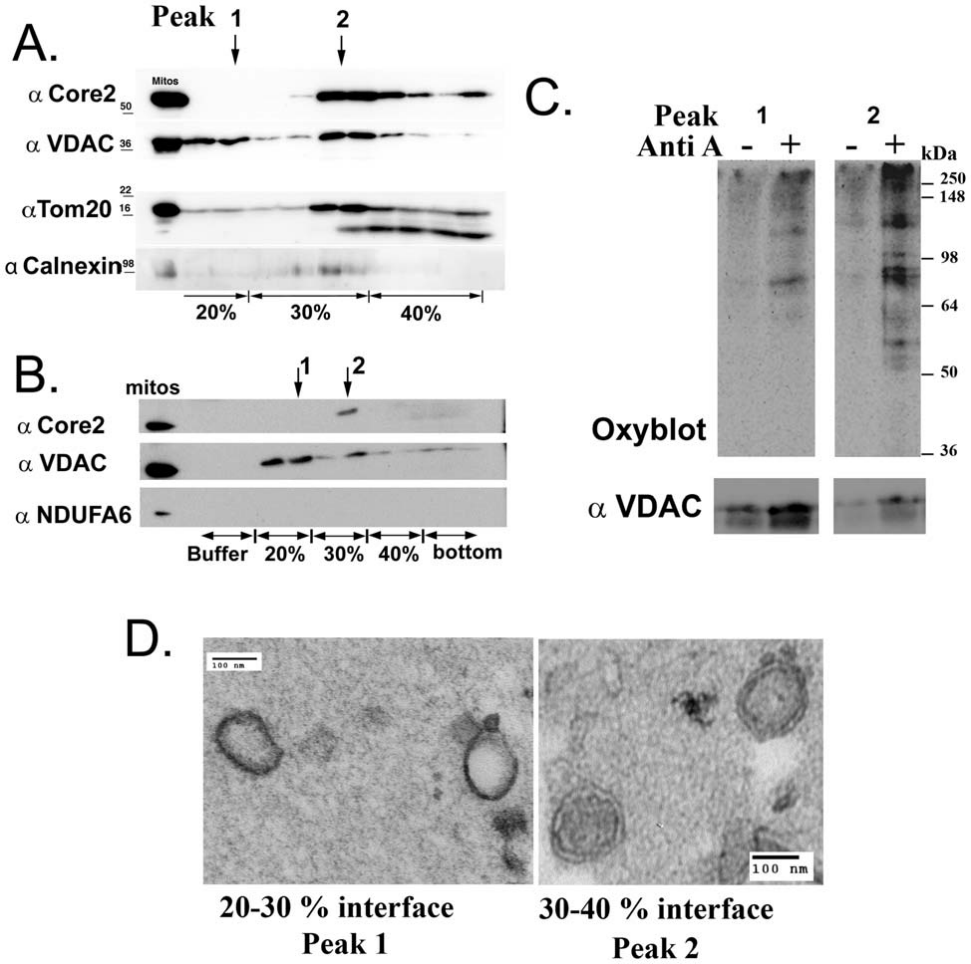
Ultrastructural analysis of MDVs generated in vitro. A) Supernatants obtained from reactions treated with Antimycin A were fractionated on a discontinuous sucrose gradient. After centrifugation, fractions were collected and analyzed by western-blot as indicated. 5 µg of starting mitochondria is included in the first lane. Two peaks of VDAC were noted, at the 20/30% interface and the 30/40% interface, as indicated by the arrows and numbers 1 and 2 above the figure. The heavier fractions also contained the inner membrane subunit Core2 of complex III. Tom20 was also seen in both fractions, although was less abundant within the lighter peak relative to VDAC (which was equally present in both peaks). The ER marker calnexin reveals some contamination in the heavier fractions. B) A repeat of A) probed for another subunit (NDUFA6) of Complex I, confirming it's absence within MDVs. Again, note the two peaks of VDAC, and Core2 present only in the heavier peak. C) Budding reactions were performed under either basal conditions, or in the presence of 50 µM Antimycin A. Following separation by sucrose density centrifugation as in A, B, proteins within peak fractions 1 and 2 were derivitized with DNPH, and conjugated proteins were separated by SDS-PAGE and revealed using anti-DNPH Oxyblot antibodies. The presence of VDAC in both peaks was used as an internal loading control. D) EM analysis from fractions in both peaks of a reaction performed in the presence of 50 µM Antimycin A was performed on fixed and low-melt agarose embedded samples. Scale bars are 100 nm.

We next wanted to further isolate the MDVs using sucrose density centrifugation in order to examine additional cargo without requiring an incubation with trypsin. Floated vesicles would separate from any broken structures, which would remain at the bottom of the gradient. In addition, the sucrose gradient would allow us to separate the single from double membrane bound vesicles for further analysis. All vesicles generated by Antimycin A treatment were first separated from intact organelles by centrifugation. This supernatant fraction was adjusted to 40% sucrose, and loaded at the bottom of a discontinuous sucrose gradient. A light fraction containing VDAC appeared at the 20/30% sucrose interface (Figure 4A, B, arrow labeled 1), and a heavier fraction containing both Core2 and VDAC sedimented at the 30/40% interface, (Figure 4A, B, arrow labeled 2). Without the need for a trypsin treatment, we examined the presence of Tom20, and an ER membrane protein calnexin (Figure 4A). Tom20 showed signs of degradation within the 40% sucrose fraction loaded at the bottom of the gradient, but the full-length protein was apparent predominantly in the heavier fraction 2, but there was some incorporation of Tom20 within the lighter fraction at 20%/30% sucrose. Calnexin was present within fraction 2, consistent with the difficulty in isolating mitochondria devoid of ER. Importantly, both fractions lacked the complex I protein NDFUA6 (Figure 4B), consistent with the cargo selectivity observed within the trypsinized supernatants shown in Figure 3A. The visualization of selective mitochondrial proteins in two distinct peaks of the sucrose gradient further demonstrates that the MDV fractions are not a result of broken mitochondria or differential trypsin sensitivity.

We then compared the oxidation levels of the peak fractions from reactions performed in basal conditions or in presence of Antimycin A. Oxidation levels of these fractions were measured using the Oxyblot system (Figure 4C) and showed that the highly purified, density separated vesicles are also enriched for oxidized cargo upon inhibition of complex III. In the absence of Antimyin A, there is very little oxidized proteins found in either of the two fractions, further supporting the specificity of the reaction. Only in the presence of Antimycin A do we observe a significant enrichment of oxidized proteins in both peaks 1 and 2 (Figure 4C). Finally, ultrastructural analysis of the light membrane fractions isolated at 20/30% sucrose confirmed the presence of single membrane vesicles, and the heavy membrane fraction isolated at 30/40% sucrose revealed the presence of double membrane bound vesicles (Figure 4D). These data directly demonstrate that the proteins found within the supernatants of the budding reaction are contained within regular, membrane bound vesicle structures of specific buoyant densities.

## Discussion

The data presented here document the exquisite specificity of cargo incorporation into mitochondrial derived vesicles. We have directly demonstrated that oxidized proteins are ejected from mitochondria in a tightly controlled, vesicular budding process. The central conclusions from this study are that the cargo incorporation into MDVs is 1) highly selective, 2) can incorporate either one or both mitochondrial membranes, and 3) are enriched for oxidized protein. The data show that MDVs incorporate subunits of complexes II, III and IV, yet complex I, V and mtDNA nucleoids are excluded. The exposure to ROS generated outside mitochondria leads to vesicles enriched for VDAC, but lacking complex III. The exclusion of the large respiratory complexes I and V, as well as mtDNA, could be due to size limitations, or perhaps they may be incorporated with different triggers not yet tested here. Also, we cannot exclude that other cell types may selectively incorporate complexes I, V and nucleoids. However, the average size of a nucleoid has been shown to be almost 70 nm [28], which could be too large for this pathway. Despite the differential cargo incorporation, MDVs appear to be of uniform size and density, as indicated by the ultrastructural analysis and from the distinct sedimentation peaks identified on sucrose gradients.

Quantitatively, the amount of protein incorporated into the vesicles *in vitro* is within the same range as the amount of proteins degraded by the yeast mitochondrial proteases per hour [2]. Consistent with previous data, our biochemical studies also show that even under basal conditions, in absence of acute stress, some vesicles carrying oxidized proteins are removed from mitochondria (Figure 3D), indicating that this is an ongoing process to maintain mitochondrial integrity [27]. This result demonstrates that mitochondria generate MDVs under respiring conditions, which we have shown previously to be cleared in the lysosome [27]. Taken together, MDV delivery of mitochondrial cargo to the lysosomes represents a significant new process for mitochondrial quality control. Unlike the requirement for global mitochondrial depolarization and dysfunction studied with CCCP treatments, we consider that MDV formation would act as an upstream mechanism of mitochondrial quality control, removing lipid and protein depending on the oxidative load.

The precise mechanism controlling the generation of stress-induced MDVs is still unknown, however our first data indicate that it can be regulated by cytosolic factors and is dependent on the flux of electrons through the respiratory chain. Similar to several budding processes it can be inhibited by the alkylation of factors containing free thiol groups by NEM, or activated by non-hydrolysable forms of GTP [36], [37]. This later result confirms that the generation of MDVs is independent of the hydrolysis of GTP, excluding the implication of the mitochondrial fission protein DRP1. Instead, it suggests the involvement of additional GTP-binding factors, perhaps similar to the Arf or Rab family of GTPases, in MDV formation.

The mitochondria generate MDVs to target proteins to at least two intracellular destinations, the peroxisomes and the lysosomes [25], [26], [27]. In this work, we have focused only on the initial budding steps, and not the target destination. There are many questions to be answered concerning whether each destination uses distinct machinery, and how the MDVs target their final destination. With this assay in hand, future work will focus on dissecting the many biochemical steps necessary to enrich protein cargo, deform the membranes, generate outward curvature, pinch the vesicle for release from the mitochondria, and tether with the acceptor organelle for the final cargo delivery.

## Supporting Information

Figure S1 MDVs contain selected mitochondrial cargo. A) Further *in vitro* mitochondrial budding reactions were performed, probing the trypsinized supernatant fractions with additional subunits of Complex 1 (NDUFS3), and for aconitase. Neither of these two proteins was enriched within the MDV fractions. B) Another set of budding reactions here reveals the absence of the nucleoid protein TFAM, consistent with the inability to PCR amplify mtDNA encoded Cox1 from the MDV fractions.
(JPG)
