# Supplementary figures and images for: Reconstitution of Mitochondria Derived Vesicle Formation Demonstrates Selective Enrichment of Oxidized Cargo
Source: PLoS One. 2012 Dec 26;7(12):e52830. doi: 10.1371/journal.pone.0052830 (PMC3530470; doi:10.1371/journal.pone.0052830)

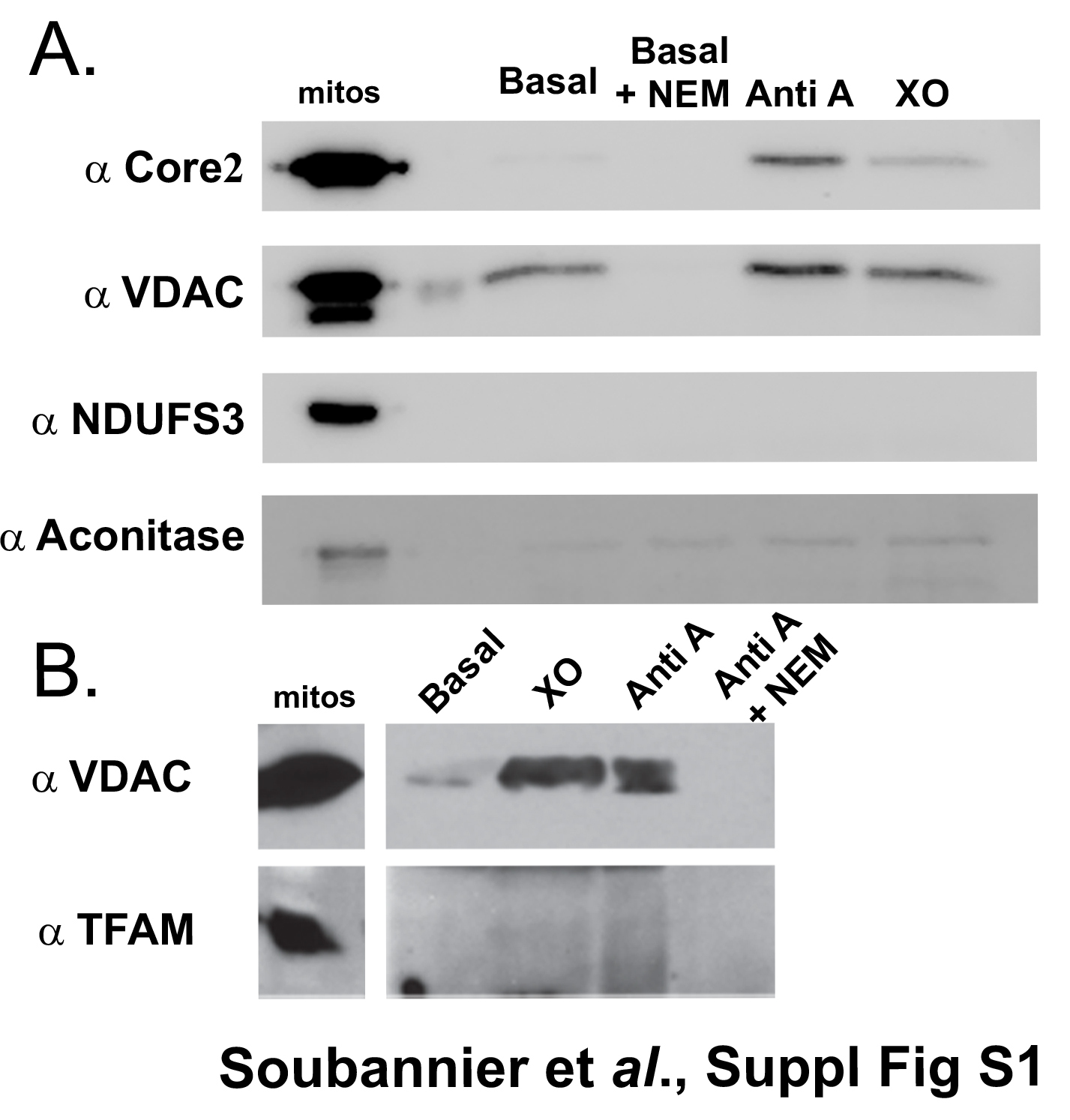

Supplement: Figure S1 — MDVs contain selected mitochondrial cargo. A) Further in vitro mitochondrial budding reactions were performed, probing the trypsinized supernatant fractions with additional subunits of Complex 1 (NDUFS3), and for aconitase. Neither of these two proteins was enriched within the MDV fractions. B) Another set of budding reactions here reveals the absence of the nucleoid protein TFAM, consistent with the inability to PCR amplify mtDNA encoded Cox1 from the MDV fractions. (JPG) [file pone.0052830.s001.jpg]
